# Delay discounting differences in brain activation, connectivity, and structure in individuals with addiction: a systematic review protocol

**DOI:** 10.1186/s13643-017-0537-0

**Published:** 2017-07-11

**Authors:** Max M. Owens, Michael T. Amlung, Steven R. H. Beach, Lawrence H. Sweet, James MacKillop

**Affiliations:** 1 Department of Psychology, University of Georgia, 125 Baldwin Street, Athens, GA 30602 USA; 2 Peter Boris Centre for Addiction Research, St. Joseph’s Healthcare Hamilton/McMaster University, 100 West 5th Street, Hamilton, ON L8P 3R2 Canada; 3 Department of Psychiatry, Warren Alpert Medical School of Brown University, Providence, RI USA

**Keywords:** Delayed reward discounting, Temporal discounting, Magnetic resonance imaging, Functional magnetic resonance imaging, Addictive behavior, Substance use disorder, Compulsive gambling

## Abstract

**Background:**

Delayed reward discounting (DRD), the degree to which future rewards are discounted relative to immediate rewards, is used as an index of impulsive decision-making and has been associated with a number of problematic health behaviors. Given the robust behavioral association between DRD and addictive behavior, there is an expanding literature investigating the differences in the functional and structural correlates of DRD in the brain between addicted and healthy individuals. However, there has yet to be a systematic review which characterizes differences in regional brain activation, functional connectivity, and structure and places them in the larger context of the DRD literature. The objective of this systematic review is to summarize and critically appraise the existing literature examining differences between addicted and healthy individuals in the neural correlates of DRD using magnetic resonance imaging (MRI) or functional magnetic resonance imaging (fMRI).

**Methods:**

A systematic search strategy will be implemented that uses Boolean search terms in PubMed/MEDLINE and PsycINFO, as well as manual search methods, to identify the studies comprehensively. This review will include studies using MRI or fMRI in humans to directly compare brain activation, functional connectivity, or structure in relation to DRD between addicted and healthy individuals or continuously assess addiction severity in the context of DRD. Two independent reviewers will determine studies that meet the inclusion criteria for this review, extract data from included studies, and assess the quality of included studies using the Grading of Recommendations Assessment, Development and Evaluation framework. Then, narrative review will be used to explicate the differences in structural and functional correlates of DRD implicated by the literature and assess the strength of evidence for this conclusion.

**Discussion:**

This review will provide a needed critical exegesis of the MRI studies that have been conducted investigating brain differences in addictive behavior in relation to healthy samples in the context of DRD. This will provide clarity on the elements of neural activation, connectivity, and structure that are most implicated in the differences in DRD seen in addicted individuals.

**Systematic review registration:**

PROSPERO CRD42017056857

**Electronic supplementary material:**

The online version of this article (doi:10.1186/s13643-017-0537-0) contains supplementary material, which is available to authorized users.

## Background

Behavioral economic research has become a key tool in the study of decision-making and has been successfully employed to better understand health behaviors and psychopathology [1–3]. Delayed reward discounting (DRD), the degree to which a future reward is discounted relative to an immediate reward, is often measured by a series of choices between smaller rewards that are immediately available and delayed rewards available at some time in the future, with money being the most commonly used reward [4]. In such a paradigm, greater devaluation of the delayed reward are conceptualized as indicating greater impulsivity of decision-making and a preference for immediate gratification [5] and as a form of impatience [6]. Elevated DRD has been consistently associated with problematic outcomes, including obesity [7], attention deficit hyperactivity disorder [8], and suicidality [9]. But, perhaps the health behavior to which DRD paradigms have been used most extensively with the greatest success is in understanding the maladaptive decision-making that accompanies addictive behaviors. Meta-analyses have found that DRD is robustly elevated in individuals engaging in addictive behaviors and highly elevated in those with an addictive disorder [10, 11] and impulsive DRD has been demonstrated to predict future engagement in addictive behaviors, such as relapse in smoking cessation treatment [12, 13]. DRD has been suggested as a trans-disease process that links addiction with other disorders and problematic outcomes that include maladaptive decision-making [14]. This makes understanding the biological processes underlying DRD in clinical populations a vital goal in order to understand the nature of this dysregulated decision-making preference and to ultimately developing treatments that can reduce morbidity associated with addiction and other disorders.

To investigate the neurobiological processes that allow individuals to make decisions between smaller immediate and larger delayed reward, a substantial number of studies have been conducted testing the neuroanatomical and neurophysiological bases of DRD using a variety of neuroimaging methods. Meta-analytic studies have found specific neural networks to be activated by DRD tasks, including networks involved in cognitive control, reward valuation, and future planning [15–17]. Additionally, studies using other methodologies have found that the connectivity of regions within these networks and their neurostructural integrity are salient to determining individual’s impulsiveness of discounting (e.g., [18–20]). A recent model integrating these findings describes the neural process of DRD decision-making as being an emergent property of these neural networks interacting with each other, as well as sensory and motor systems, in recursive loops to use past experiences to assess the subjective value of the two options, compare them, and respond appropriately based on that comparison [21].

While individual studies have explored how these neural correlates differ in individuals with addictive disorders, the aggregated literature has yet to be summarized and systematically interpreted. Thus, a systematic investigation and evaluation of these studies is needed to determine what conclusions can and cannot be drawn regarding the differences in the neural correlates of DRD in addicted populations. Given findings of reliable network activity during DRD tasks and maladaptive patterns of DRD decision-making in addicted individuals, we hypothesize that individuals with addictive disorders will exhibit significant differences in neural activity in the networks used in intertemporal decision-making while making these decisions, systematic differences in connectivity within and between these networks during a DRD task and at rest, and diminished structural integrity in these networks. Furthermore, we hypothesize that these differences will be associated with greater impulsiveness of discounting.

## Objectives

The objective of this systematic review is to summarize the existing literature examining differences between addicted and healthy individuals in the neural correlates of DRD using magnetic resonance imaging (MRI) or functional magnetic resonance imaging (fMRI). To do so, we will examine differences in neural activation during DRD tasks, the relationship of brain connectivity during a DRD task and during rest to DRD, and the relationship of neurostructural integrity to DRD.

Specifically, our aims are to:

1. Summarize the primary peer-reviewed empirical research using neuroimaging to examine brain differences impacting DRD decision-making between individuals with addictive disorders and healthy individuals. Included in the definition of neuroimaging methods, we will include MRI and fMRI. Included in the definition of addictive disorders, we will include individuals exhibiting symptoms of addictive disorders included in the Diagnostic and Statistical Manual of Mental Disorder: fifth edition (DSM5; [22]). Included in the definition of addictive disorders will be individuals exhibiting symptoms of nicotine, alcohol, or drug use disorders and of gambling disorder as defined by the DSM5. Specific outcome measures to be reviewed include regional neural activation during a DRD task, functional connectivity among brain regions during a DRD task and at rest, and regional gray or white matter volume, thickness, or area.
2. Critically appraise the current state of the literature and determine gaps that require further study.

## Methods

### Search strategy

Existing primary research will be identified and retrieved using MEDLINE/PubMed and PsycINFO. Relevant articles will be identified using a comprehensive search strategy for all terms related to DRD and neuroimaging (Table 1). To identify relevant articles that were not captured by our initial search strategy, we will also conduct a manual search of the citations of included studies and the other works of authors of included studies.

**Table 1:** Search strategy for identifying articles on neuroimaging correlates of delayed reward discounting

1. delay*	16. tobacco
2. time	17. nicotine
3. *temporal	18. smok*
4. monetary	19. marijuana
5. reward	20. cannabis
6. discounting	21. opiate
7. choice	22. opioid
8. decision making	23. heroin
9. “impulsive choice”	24. cocaine
10. “delay gratification”	25. *amphetamine
11. functional magnetic resonance imaging	26. drug
12. magnetic resonance imaging	27. substance use
13. neuroimaging	28. substance abuse
14. alcohol*	29. gambling
15. cigarette	30. addicti*
31. DRD SEARCH TERMS	((1 OR 2 OR 3 OR 4 OR 5) AND (6 OR 7 OR 8)) OR 9 OR 10
32. MRI SEARCH TERMS	11 OR 12 OR 13
33. ADDICTIVE BEHAVIOR SEARCH TERMS	14 OR 15 OR 16 OR 17 OR 18 OR 19 OR 20 OR 21 OR 22 OR 23 OR 24 OR 25 OR 26 OR 27 OR 28 OR 29 OR 30
34. FINAL SEARCH TERMS	31 AND 32 AND 33

### Inclusion/exclusion criteria

This review will include published peer-reviewed neuroimaging studies that investigate differences in brain activation, functional connectivity, or structure between individuals with symptoms of addictive disorders and healthy individuals in the context of DRD. Included studies will include a DRD task, examine human subjects, include MRI or fMRI methodology, and include a direct comparison between an addictive behavior group and healthy group or include a continuous analysis of addictive behavior severity with at least some subjects exhibiting significant addictive disorder symptomology. To maximize the depth of the review and focus on a set of more homogenous studies, no neuroimaging methods other than MRI and fMRI will be included. No restrictions will be placed on the type of reward used in the DRD task (i.e., monetary rewards, drug rewards, and other rewards are acceptable). No age or other demographic restrictions will be used. Studies will be excluded which make inferences about brain function or structure without using neuroimaging methods to assess these inferences.

### Outcome measures

Outcome measures for this review will be the assessment of brain function as it relates to DRD measured by fMRI and assessment of brain structure as it relates to DRD measured by MRI. Three different outcome measures will be obtained from these imaging modalities: (1) regional brain activation during DRD tasks, (2) functional connectivity between brain regions during DRD tasks or the relationship of DRD rate to functional connectivity “at rest,” and (3) the relationship of DRD rate with regional gray matter or white matter volume, surface area, or thickness. These three outcome measures will each be assessed for differences between addicted and not-addicted samples if the existing literature allows.

### Data management

Resulting articles from the initial search will be uploaded to Covidence, an online systematic review management system. Training will be provided to all authors on this software. When title and abstract screening is complete, full-text articles will be uploaded to Covidence for review.

### Selection of studies

Two reviewers (MO and MA) will independently complete an initial title and abstract screening to identify eligible articles using a predetermined criterion, and any disagreements between the two reviewers will be settled by a third reviewer (JM). Following title and abstract screening, articles that are determined to be eligible will be retrieved and full-text reviewed. In this phase, studies determined to be ineligible will be excluded from the review. Reasons for exclusion will be reported using the Preferred Reporting Items for Systematic Reviews and Meta-Analyses (PRISMA) flow diagrams. At all phases of screening, kappa values will be calculated to assess inter-rater agreement with kappa values over 0.75 considered as high agreement.

### Review procedures

The two reviewers will also independently record information from the studies included in the review in separate electronic spreadsheets. A calibration exercise will be performed to maximize consistency. The following information will be recorded from each study: publication details (publication names, authors, year of publication, journal, and country), imaging modality, satisfaction of inclusion/exclusion criteria, data analysis strategy used, sample size, demographic characteristics of sample (age, ethnicity, sex), behavioral measures of DRD task performance (e.g., *k* values, area under the curve), whether the DRD task was completed in or out of scanner, the choice coding strategy used to generate fMRI contrasts (e.g., impulsive vs. restrained choices), addiction severity (i.e., clinical diagnosis given, validated self-report measure of severity, or quantity/frequency of involvement), neuroimaging data analysis strategy used, and any outcome measure pertaining to DRD assessed in an included study (i.e., regional brain activation, regional gray matter volume, functional connectivity among regions). For outcome measures, test statistics, *p* values, and effect sizes will be recorded. For studies in which one or more of these metrics is unavailable, the metric will be calculated if possible or the authors of the study contacted if calculation from existing data is not possible. Additionally, it will be recorded if comorbid mental disorders or comorbid addictive behaviors were present and if they were an exclusion criteria or used as a matching variable between experimental and control groups.

### Assessment of quality

Two independent reviewers will assess overall quality of evidence using the criteria outlined in the Grading of Recommendations Assessment, Development and Evaluation (GRADE) framework, examining quality of the literature in five domains—risk of bias, publication bias, consistency, directness, and precision [23]. A rating in each domain and overall will be given for each outcome measure being investigated. Examples of study design features that will indicate high-quality studies include large sample size (100 or greater considered high quality, between 20 and 99 considered moderate quality, fewer than 20 considered low quality), adequate assessment of addiction severity (clinical diagnosis considered highest quality, self-report assessment of addiction severity considered moderate quality, quantity/frequency measures of substance use/gambling considered low quality), assessment of and accounting for comorbid mental health disorder or engagement in multiple addictive behaviors either by exclusion or sample matching, use of a high-quality and/or previously validated DRD paradigm, use of accepted and validated indices of DRD (e.g., *k*, area under the curve), and high-quality MRI and fMRI methods (e.g., 1.5 vs. 3 T MRI). Additionally, neuroimaging analysis design will be considered as a factor in study quality, whole-brain analyses using a strategy to sufficiently control type I error being considered high quality, region of interest analysis using sufficient type I error control strategies considered moderate quality, and insufficiently whole-brain or region of interest studies with insufficient accounting for type I error considered lower quality. In this definition the term “whole-brain” will refer to voxel-by-voxel analysis, atlas-based analyses examining all regions of the brain, functional connectivity between nodes in all regions of the brain, or statistical data reduction strategies used across the entire brain (e.g., independent component analysis or multi-voxel pattern analysis). The term “region of interest” will refer to analyses looking at brain activation or structure in a limited number of regions of the brain, using a voxel-by-voxel approach in a limited segment of the brain (e.g., frontal lobe only or using disjunction mask approach), or using a limited number of functional connectivity nodes chosen based on a priori or empirical methods.

### Strategy of data synthesis

Narrative review will be used to explicate the specific findings within the domain of each of the three outcome measures: brain activation during DRD task fMRI, functional connectivity during DRD task or resting fMRI, and brain structure via MRI. For each outcome measure, we will evaluate evidence for and against the conclusion that there are differences between addicted and healthy populations. The strength of evidence for this conclusion will be determined by the items assessed in the GRADE framework described above. Areas needing further study will be highlighted, and suggestions for future study will be made.

Since previous meta-analytic studies have found larger differences in discounting in clinically diagnosed individuals compared to undiagnosed individuals [10] and larger associations between discounting and clinical severity compared to quantity-frequency measures [11], if possible from number of studies available, we will use narrative review to explicate if a “dose-dependent” relationship can be identified between brain abnormality pertaining to DRD and addictive behavior. To do this, we will consider differences in task activation, functional connectivity, and brain structure pertaining to DRD at the three levels of addiction severity assessment resolution (i.e., diagnosis, self-report severity, quantity/frequency).

### Presenting and reporting of results

This protocol follows the guidelines outlined in the Preferred Reporting Items for Systematic Reviews and Meta-Analysis Protocols (PRISMA-P) statement [24], and the PRISMA-P 2015 checklist for this protocol is available as a document file in Additional file 1. The review itself will follow the PRISMA guidelines of reporting systematic reviews [25]. A flow chart documenting the article selection process and reasons for exclusion will be compiled, and study characteristics will be compiled in summary tables. Details of individual studies will be listed in table format with separate sections for task activation fMRI studies, fMRI connectivity studies, and structural MRI studies. A synopsis of the quality of the literature for each of these outcome measures will be provided in a separate table.

## Discussion

We expect that the results of this systematic review will provide a much needed critical exegesis of the MRI studies that have been conducted investigating brain differences in addictive behavior and healthy samples in the context of DRD. Our hope is that this will provide clarity on the elements of neural activation, connectivity, and structure that are most implicated in the differences in DRD seen in addicted individuals. To our knowledge, this will be the first systematic review conducted on this topic. Given the increased importance of DRD in understanding the neural bases of the maladaptive decision-making that characterizes substance use and gambling disorders, this review fills a need for addictive behavior and decision-making researchers.

## Additional file

Additional file 1: Preferred Reporting Items for Systematic Reviews and Meta-Analysis Protocols (PRISMA-P) checklist. This file is in .docx format and contains specific line and page numbers at which this manuscript addresses each of the criteria required by the PRISMA-P guidelines. (DOCX 37 kb)

## Abbreviations

DRD: Delayed reward discounting
DSM5: Diagnostic and Statistical Manual of Mental Disorders: fifth edition
fMRI: Functional magnetic resonance imaging
GRADE: Grading of Recommendations Assessment, Development and Evaluation
MRI: Magnetic resonance imaging
PRISMA: Preferred Reporting Items for Systematic Reviews and Meta-Analyses
PRISMA-P: Preferred Reporting Items for Systematic Reviews and Meta-Analysis Protocols
